# A systematic review and meta-analysis of adherence to physical activity interventions among three chronic conditions: cancer, cardiovascular disease, and diabetes

**DOI:** 10.1186/s12889-019-6877-z

**Published:** 2019-05-24

**Authors:** Tiffany Bullard, Mengmeng Ji, Ruopeng An, Linda Trinh, Michael Mackenzie, Sean P. Mullen

**Affiliations:** 1 University of Illinois at Urbana-Champaign, Champaign, USA; 2 University of Toronto, Toronto, Canada; 3 University of Delaware, Newark, Delaware USA

**Keywords:** Exercise adherence, Dropout, Chronic disease, Cancer, Cardiovascular disease, Diabetes

## Abstract

**Background:**

Physical activity is effective for the prevention and treatment of chronic disease, yet insufficient evidence is available to make comparisons regarding adherence to aerobic physical activity interventions among chronic disease populations, or across different settings.

The purpose of this review is to investigate and provide a quantitative summary of adherence rates to the aerobic physical activity guidelines among people with chronic conditions, as physical activity is an effective form of treatment and prevention of chronic disease.

**Methods:**

Randomized controlled (RCTs) trials where aerobic physical activity was the primary intervention were selected from PsychInfo, PubMed, CINAHL (Cumulative Index to Nursing and Allied Health Literature), Clinical Key, and SCOPUS from 2000 to 2018. Studies were included if the program prescription aligned with the 2008 aerobic physical activity guidelines, were at least 12 weeks in length, and included adult participants living with one of three chronic diseases. The data was extracted by hand and the PRISMA (preferred reporting items for systematic review and meta-analysis) guidelines were used to evaluate risk-of-bias and quality of evidence. Data were pooled using random-effect models. The primary outcome measure was program adherence and the secondary outcome measures were dropout and setting (e.g. home vs. clinic-based). Pooled effect sizes and 95% CiIs (confidence intervals) were calculated using random-effect models.

**Results:**

The literature search identified 1616 potentially eligible studies, of which 30 studies (published between 2000 and 2018, including 3,721 participants) met the inclusion criteria. Three clinical populations were targeted: cancer (*n* = 14), cardiovascular disease (*n* = 7), and diabetes (*n* = 9). Although not statistically significant, adherence rates varied across samples (65, 90, and 80%, respectively) whereas dropout rates were relatively low and consistent across samples (5, 4, and 3%). The average adherence rate, regardless of condition, is 77% (95% CI = 0.68, 0.84) of their prescribed physical activity treatment. The pooled adherence rates for clinic-based and home-based programs did not differ (74% [95% CI, 0.65, 0.82] and 80% [95% CI, 0.65, 0.91], respectively).

**Conclusions:**

The current evidence suggests that people with chronic conditions are capable of sustaining aerobic physical activity for 3+ months, as a form of treatment. Moreover, home-based programs may be just as feasible as supervised, clinic-based physical activity programs.

## Background

Chronic disease is the leading cause of death in America and almost 50% of adults have one or more chronic health conditions [1]. Increasing physical activity has been shown to be an effective form of treatment and prevention of chronic disease [2–4]. The benefits of regular physical activity include, but are not limited to, weight control, strengthening of muscles and bones, increases in balance and general physical functioning, and improvements in mental health [5] and health-related quality of life [6]—all factors negatively affected by chronic disease. Current public health guidelines [7] recommend 150 min of moderate-to-vigorous aerobic exercise per week. However, it has previously been reported that only 35% of women after breast cancer diagnosis [8], 32% of those with cardiovascular disease (CVD) [9], and 46% of people with diabetes met physical activity guidelines [10]. These low adherence rates are not altogether surprising, as individuals with chronic disease have many barriers (i.e. fatigue, pain) to continued physical activity participation relative to those without chronic disease.

Typical treatment for chronic disease involves managing symptoms with medication and accounts for 86% of the total health care expenses in the United States [11]. There is evidence within the medical community that physical activity is comparably effective as an additional treatment of disease [12] and lowering the risk of mortality [13] relative to standard treatment methods (e.g. medications, surgery, chemotherapy and radiation). However, research trials can vary substantially in their methodologies as well as their setting (clinic- vs. home-based). Both settings have unique advantages. Clinic-based programs often provide more detailed and intensive supervision, whereas home-based programs typically provide more autonomy (e.g., more choices regarding training schedule, fewer transportation-related barriers to receive intervention). In a review of physical activity interventions designed for older adults, Conn et al. [14] found a greater effect for clinic-based interventions (*d* = .26) relative to home-based programs. It is possible that the supervision provided in the clinic-based studies resulted in greater adherence to the program. It cannot be assumed that patients will uniformly adhere to any structured physical activity program, irrespective of their condition. Specifically, do patients’ adherence levels vary across chronic conditions (e.g., cardiovascular vs. metabolic) or type of program (e.g., clinic or home-based)? Answering these questions is essential for practitioners and researchers, as both are interested in understanding how to optimize the delivery of physical activity as medicine as an adjuvant treatment for disease.

The three most commonly studied chronic diseases in the context of physical activity interventions are cancer, CVD, and diabetes. Physical activity has been shown to be an effective treatment for each of these diseases and current evidence suggests exercise has a positive effect on patient quality of life, physical functioning, and fatigue compared to usual care. For example, Gerritsen and Vincent [15] examined the evidence from randomized controlled trials involving cancer patients in a systematic review and meta-analysis and determined that exercise significantly improved self-esteem, physical performance and functioning, fatigue, and social functioning. According to an observational study of 2987 women diagnosed with breast cancer [16], those who participated in regular physical activity (9+ MET (metabolic equivalent task)-hours per week) saw reductions in breast cancer mortality (relative risk: 0.50, 95% CI: 0.34–0.74). Similarly, Anderson et al. [17] reviewed exercise-based cardiac rehabilitation programs and, relative to usual care, exercise improved quality of life, reduced hospital admissions post-treatment (relative risk: 0.82, 95% CI: 0.70 to 0.96), and reduced cardiovascular mortality (relative risk: 0.74, 95% CI: 0.64 to 0.86), independent of study quality, setting, and publication date. Likewise, Umpierre et al. [18] provided substantial evidence that structured exercise training is associated with reduced levels of hemoglobin A_1C_ (HbA_1C_) (− 0.67%; 95% CI: -0.84% to − 0.49%, *p* < 0.001), as well as reduced risks for diabetes-related complications in patients with type 2 diabetes. Hu et al. conducted a longitudinal study of 3708 patients with type 2 diabetes [19] and showed a reduction in mortality risk across low, moderate, and high physical activity levels (relative risk: 1.00, 0.59, and 0.49, respectively). Altogether, there is substantial evidence for using exercise as medicine [20] (i.e. treating disease, lowering mortality), but comparisons regarding adherence to aerobic exercise prescriptions across these conditions, and between clinic- and home-based settings, have not been made. Given that exercise can serve as a standalone and complementary medicine, more research is needed examining the relative acceptability of activity prescriptions across populations.

In this systematic review and meta-analysis, we aimed to test the potential differences in adherence and dropout rates among patients involved in aerobic physical activity interventions. We hypothesized that cancer patients would exhibit the lowest adherence rates with the knowledge that very few cancer patients meet the recommended physical activity guidelines (2008 or 2018) for aerobic exercise, and given the long-lasting and debilitating effects of chemotherapy and radiation treatment (e.g., fatigue, cognitive impairment) [21] compared to CVD or diabetes. In addition, we also hypothesized higher adherence associated with clinic-based programs relative to home-based programs because there is arguably more supervision and accountability in such programs.

## Methods

### Study inclusion criteria

RCTs with an aerobic (only) exercise intervention were included in the review. Specifically, trials must have included an explicit program prescription aligned with the 2008 Physical Activity Guidelines for aerobic exercise (i.e., a minimum of 150 min per week). Also, trials that included adult participants (age 18+), and published results met study inclusion criteria. Physical activity interventions lasting at least 12 weeks were included to align with expected dropout trends previously reported in the literature among people with and without clinical conditions [22, 23]. Studies were included if rates of adherence and dropout were explicitly reported. Author confirmation was required if study data was not reported in sufficient detail. In this study, adherence was defined as *meeting the aerobic physical activity recommendations of 150 min/week across the study duration* (expressed as a percentage). Dropout was defined as *participants who formally withdrew or left the study and did not return (*e.g. *non-responders who did not officially relinquish their consent to participate).* This threshold was determined as one month or longer as a substantial period of time, whereas less than one month could reflect brief illness or vacation. If the paper provided definitions that varied from the ones above, or did not explicitly provide values, the author was contacted and asked to provide the information as requested in order to standardize the data. The intervention frequency, intensity, type, and duration were recorded for each study and each intervention was identified as either home- or clinic-based. Inclusionary criteria allowed for all types of cancer, CVD, and diabetes, as long as patients were currently diagnosed with one of the targeted diseases. Although type-1 diabetes was not exclusionary, all diabetes studies in this review included populations with type-2 diabetes. The type of cancer patients’ treatment (e.g. chemotherapy, radiation, surgery) was not exclusionary.

### Study exclusion criteria

Studies were excluded for a variety of reasons. The most common reason for exclusion was that exercise was not the primary intervention. For example, a lifestyle intervention study [24] provided counseling with the primary outcome being weight loss. Although physical activity was assessed within the study, it was not a primary outcome of the intervention. Exclusionary criteria did not allow for healthy populations (studies that did not include patients with cancer, CVD, or diabetes), or if the patients did not currently have the disease (e.g., cancer survivors).

### Search strategy

The research databases PsychInfo, PubMed, CINAHL, Clinical Key, and SCOPUS were searched limiting the publication range from 2000 to 2018. The keywords were ‘physical activity’ and ‘exercise,’ with ‘adherence,’ ‘compliance,’ or ‘drop out’ for ‘cancer’, ‘cardiovascular disease,’ ‘coronary disease,’ ‘coronary risk,’ or ‘diabetes’. Filters were used to select only RCTs when available. Additional studies were added through a manual search targeting existing meta-analyses and systematic reviews of physical activity interventions for specific clinical populations. Experts within the field were contacted for any published papers the authors may have omitted.

### Study selection

**Fig. 1:**
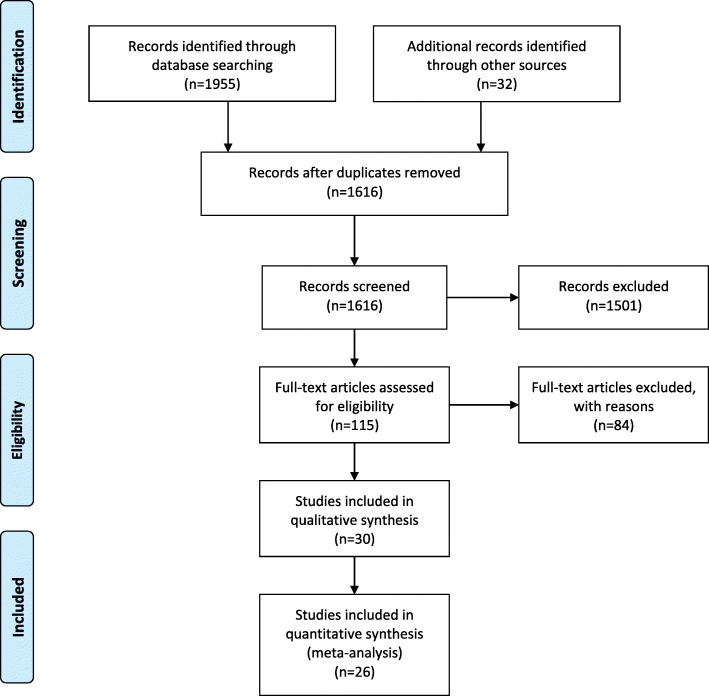
PRISMA Flow Diagram

**Fig. 2:**
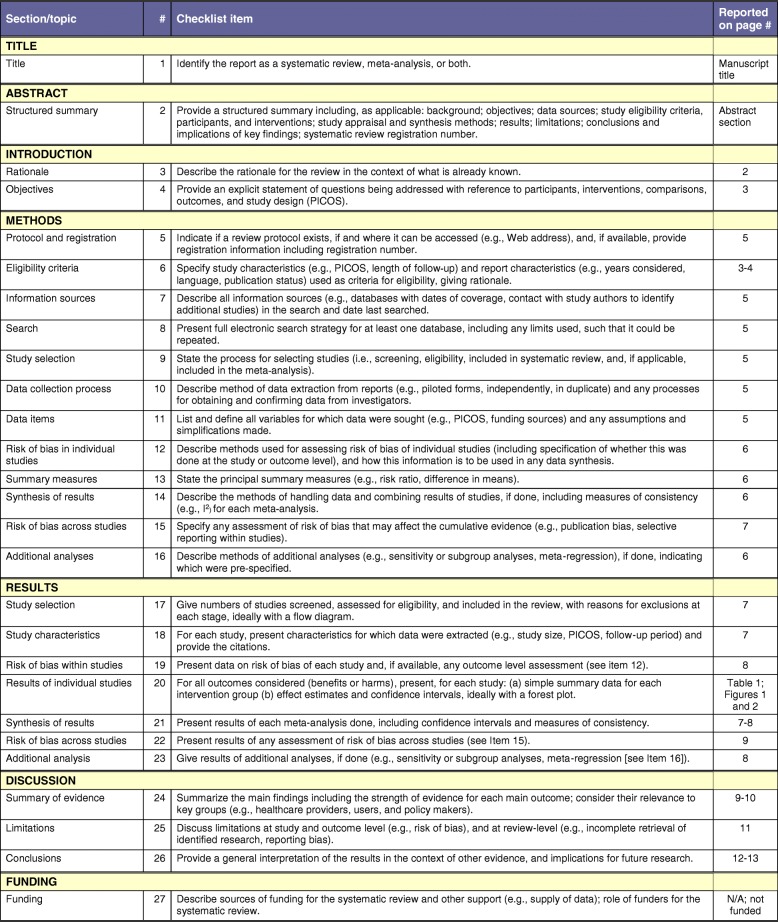
PRISMA Checklist

The PRISMA-P (preferred reporting items for systematic review and meta-analysis protocols) guidelines [25] were followed in the reporting of this systematic review protocol (see Figs. 1 and 2). The search was conducted from February 2016 to October 2018. Titles and abstracts containing the key words were searched more thoroughly to ensure the selection criteria was met. Articles needed to be written in English and included adult samples only to be considered for the review. The included and excluded publications were subsequently reviewed by a second author (S. P. M.) until 100% consensus was reached regarding the final sample of studies to be included in the analysis. Disagreements about papers meeting all requirements were discussed amongst the authors until a consensus was reached.

### Data extraction

The data for this review and meta-analysis was extracted by hand and stored within a Microsoft Excel spreadsheet. Data were extracted by the first author and checked for alliance with search criteria by the senior author. Data included information about the publication (authors, year, title), participant characteristics (number, age, gender, disease type), intervention characteristics (home- vs. clinic-based, length), and measurement characteristics (adherence definition and rates, dropout definition and rates, confirmation of data by author, intention-to-treat analysis). Authors were contacted if a measure was not explicitly reported within the publication.

### Analysis and synthesis

A meta-analysis was performed to estimate the pooled difference in dropout rates between the intervention and control group and the pooled adherence rate in the intervention group. In addition, separate pooled effect sizes were estimated based on studies stratified by disease type (i.e., cancer, CVD, and diabetes). A random-effect model was estimated given a *p*-value less than 0.05 from the Cochran’s Q test or an I^2^ statistics greater or equal to 50%; otherwise, a fixed-effect model was estimated. Meta-regressions were conducted to examine the potential heterogeneities in differential dropout rates between the intervention and control group and adherence rate in the intervention group attributable to different disease type. The independent variables in both meta-regressions were two categorical variables for CVD and type 2 diabetes, with cancer as their common reference group. Additional meta-regressions were conducted to assess dropout/adherence rates in relation to intervention duration (measured by a continuous variable for trial length in weeks), intention to treat (ITT) status (measured by a dichotomous variable for intervention conducted following the ITT principle), age (measured by a continuous variable for mean age of the study sample), and intervention setting (measured by a dichotomous variable for home-based intervention, with clinic-based intervention as the reference group). If studies with multiple intervention groups were included in the review, only the aerobic exercise group was included included in the analysis in comparison to the usual care group.

Publication bias was assessed using the Begg’s test and Egger’s test. All analyses used two-sided tests, and *p*-values less than 0.05 were considered statistically significant. All statistical analyses were conducted using Stata 15.1 SE version (StataCorp, College Station, TX).

## Results

### Characteristics of included studies

Following a comprehensive literature search, there were 1616 eligible studies, published between 2000 and 2018. The search and selection of articles are summarized in the study flow diagram (Fig. 1). Study characteristics can be found in Table 1. The median sample size was 81 participants (range 14 to 606). A total of 3,721 participants were included in this review. The 30 studies included examined cancer (*n* = 14), CVD (*n* = 7), and diabetes (*n* = 9). All studies included patients that were currently diagnosed with any type of cancer, CVD, or diabetes. Among the cancer studies, there were four types included in this review: breast, prostate, colorectal, and ovarian. The CVD studies included heterogeneous samples (any CVD-related diagnoses) and homogeneous samples (e.g. coronary disease, heart failure, and hypertension). Although we did not exclude any specific type of diabetes, all studies within this review included patients diagnosed with type 2 diabetes. The mean age was 57.32 (*SD* = 7.40). All studies included an aerobic exercise program, meeting the 2008 and 2018 Physical Activity Guidelines of a minimum of 150 min of moderate-intensity aerobic activity per week. Half of the studies included were clinic-based (*n* = 16, 53.3%). The pooled adherence rates for clinic-based and home-based programs were 74% [95% CI, 0.65, 0.82] and 80% [95% CI, 0.65, 0.91], respectively. Across conditions, there was greater variability for the number of clinic-based programs: cancer (*n* = 7, 50.0%), CVD (*n* = 2, 28.6%), and diabetes (n = 7, 77.8%). The mean length of the intervention was 20 weeks (range 12 to 52 weeks). Of the 30 studies, 12 studies report the use of intention-to-treat (ITT) method for missing data, 13 studies report not using ITT, and 5 studies did not confirm the type of analysis used.

**Table 1:** Study Characteristics

First author, year (superscript = references)	Sample size	Duration	Chronic disease	Type	Age in years (mean)	Location
Gokal et al., 2016 [35]	50	12 weeks	cancer	breast	52.00	home-based
Huang et al., 2015 [36]	159	12 weeks	cancer	breast	48.27	home-based
Cadmus et al., 2009 [37]	50	24 weeks	cancer	breast	55.80	home-based
Courneya et al., 2009 [38]	122	12 weeks	cancer	lymphoma	53.20	clinic-based
Segal et al., 2008 [39]	81	24 weeks	cancer	prostate	65.75	clinic-based
Al-Majid et al., 2015 [40]	14	12 weeks	cancer	breast	50.30	clinic-based
Courneya et al., 2008 [41]	242	17 weeks	cancer	breast	50.00	clinic-based
Dodd et al., 2010 [42]	119	12 weeks	cancer	breast, colorectal, or ovarian	50.50	home-based
Duijts et al., 2012 [43]	422	12 weeks	cancer	breast	48.20	home-based
Giallauria et al., 2015 [44]	94	52 weeks	cancer	breast	53.50	clinic-based
Nikander et al., 2007 [45]	28	12 weeks	cancer	breast	53.00	clinic-based
Pickett et al., 2002 [46]	52	12 weeks	cancer	breast	52.00	home-based
Shang et al., 2012 [47]	126	20 weeks	cancer	breast, colorectal, or prostate	60.20	clinic-based
Courneya et al., 2003 [48]	102	16 weeks	cancer	colorectal	60.00	home-based
Lian et al., 2014 [49]	330	12 weeks	CVD	coronary artery disease	62.30	home-based
Li et al., 2015 [50]	77	12 weeks	CVD	CVD	80.68	home-based
Salvetti et al., 2008 [51]	39	12 weeks	CVD	coronary disease	53.00	home-based
Gary et al., 2011 [52]	24	12 weeks	CVD	heart failure	60.00	home-based
Guimaraes et al., 2014 [53]	32	12 weeks	CVD	hyper-tension	53.70	clinic-based
Houle et al., 2011 [54]	65	52 weeks	CVD	acute coronary syndrome	59.00	home-based
Kitzman et al., 2010 [55]	53	16 weeks	CVD	heart failure	70.00	clinic-based
Lee et al., 2015 [56]	80	12 weeks	diabetes	type 2	56.08	home-based
Church et al., 2010 [57]	113	36 weeks	diabetes	type 2	55.80	clinic-based
Dela et al., 2004 [58]	24	12 weeks	diabetes	type 2	51.50	home-based
Balducci et al., 2014 [59]	127	48 weeks	diabetes	type 2	60.00	clinic-based
Negri et al., 2010 [60]	60	16 weeks	diabetes	type 2	65.70	clinic-based
Nicolucci et al., 2012 [61]	606	49 weeks	diabetes	type 2	60.00	clinic-based
Sigal et al., 2007 [62]	251	22 weeks	diabetes	type 2	53.50	clinic-based
Nam et al., 2012 [63]	140	24 weeks	diabetes	type 2	56.39	clinic-based
Tessier et al., 2000 [64]	39	16 weeks	diabetes	type 2	69.40	clinic-based

### Dropout and adherence rates of the interventions

**Fig. 3:**
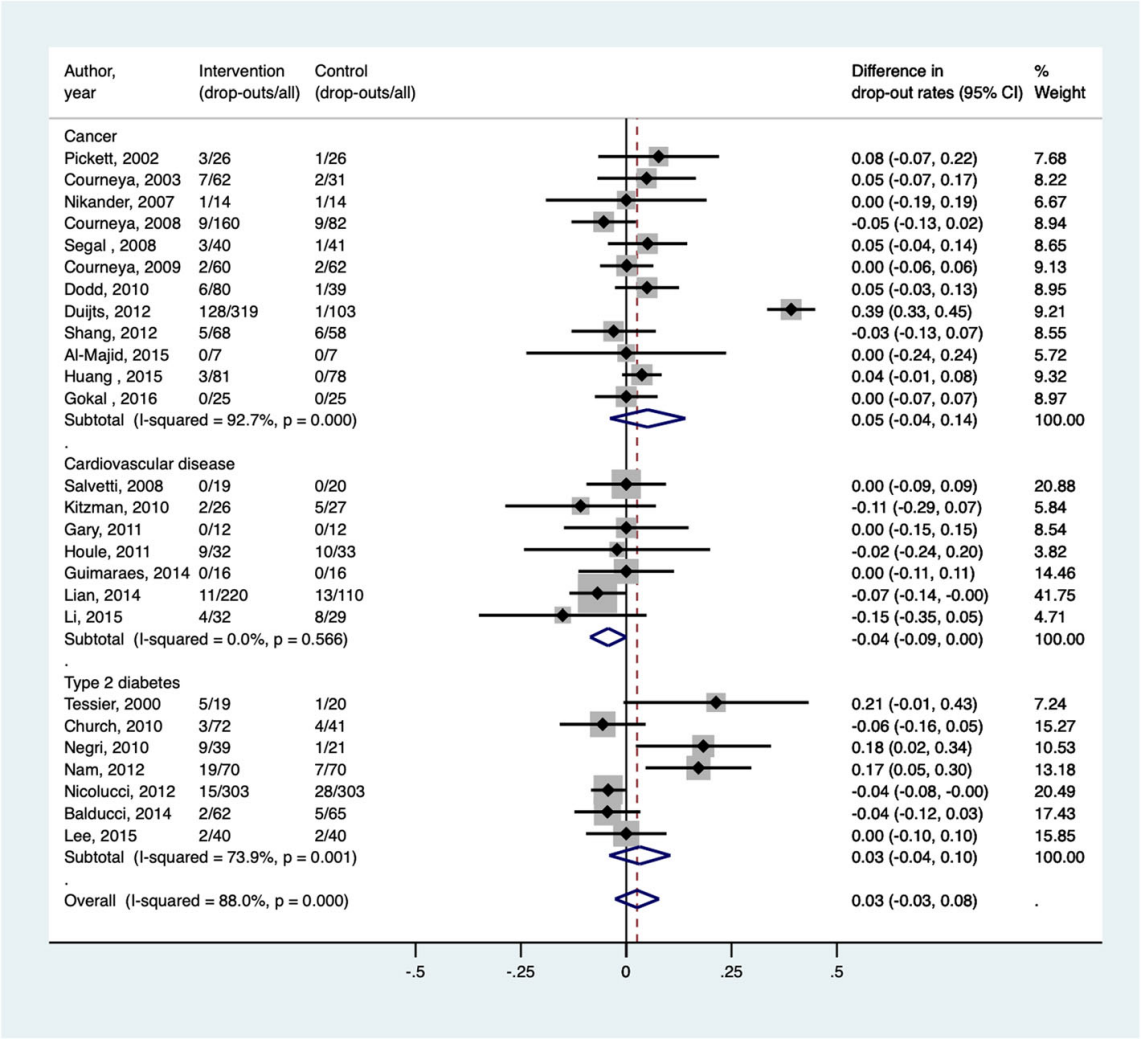
Forest plot of meta-analysis estimates on the pooled difference in dropout rates between the intervention and control group for all studies and studies stratified by disease type

Overall, the un-weighted average adherence rate was 77% and the dropout rate was 7.0%. No significant differences were found between the three chronic diseases. The pattern of data merely suggest that cancer patients had greater variability in adherence and dropout (adherence: min. = 30.1%, max. = 92.9%; dropout: min. = 0%, max. = 40.1%), compared to CVD (adherence: min. = 80.9%, max. = 100%; dropout: min. = 0%, max. = 28.1%) and diabetes (adherence: min. = 48.6%, max. = 100%; dropout: min. = 0%, max. = 27.1%). We also did not see differences in adherence rates between strictly clinic-based (74% [95% CI, 0.65, 0.82]) and home-based (80% [95% CI, 0.65, 0.91]) aerobic exercise programs. Fig. 3 reports the meta-analysis estimates on the pooled difference in dropout rates between the intervention and control group for all studies and studies stratified by disease type (i.e., cancer, CVD, and diabetes). The estimated pooled difference in dropout rates based on all studies who reported dropout rates for both the intervention and control groups (*n* = 26) was 0.03 (95% confidence interval [CI] = − 0.03, 0.08), denoting a lack of difference in dropout rates between intervention and control groups. Subgroup meta-analysis revealed no statistical significance as well. The estimated pooled differences in dropout rates based on studies with cancer (*n* = 12), CVD (*n* = 7), and type 2 diabetes (n = 7) patients were 0.05 (95% CI [− 0.04, 0.14]; I^2^ = 92.7%), 0.04 (95% CI [− 0.09, 0.00]; I^2^ = 0.0%), and 0.03 (95% CI [− 0.04, 0.10], I^2^ = 73.9%). The I^2^ did show substantial heterogeneity for dropout among the sub-diseases. Meta-regression revealed no difference in the differential dropout rates between intervention and control groups across studies stratified by disease type, intervention duration, ITT status, age, or intervention setting. The *p*-values for the Begg’s test and Egger’s test were 0.13 and 0.85, respectively, denoting a lack of publication bias.

**Fig. 4:**
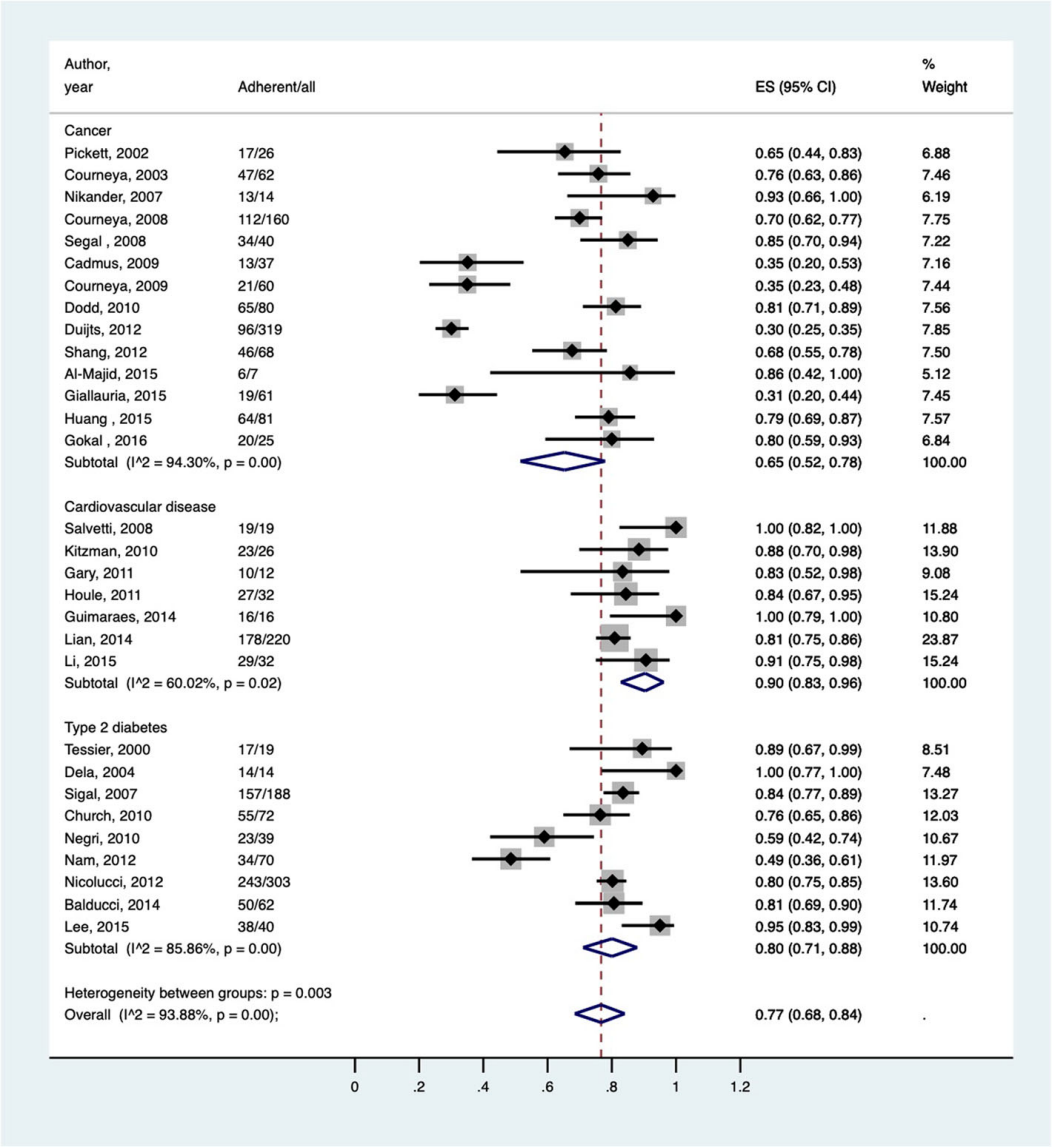
Forest plot of meta-analysis estimates on the pooled adherence rate (proportion) in the intervention group for all studies and studies stratified by disease type

Figure 4 reports the meta-analysis estimates on the pooled adherence rate in the intervention group for all studies and studies stratified by disease type. The estimated pooled adherence rate based on all studies (*n* = 30) was 0.77 (95% CI = 0.68, 0.84) in the intervention group. The estimated pooled adherence rates for patients in the intervention groups of the cancer (*n* = 14), CVD (*n* = 7), and type 2 diabetes (*n* = 9) studies were 0.65 (95% CI [0.52, 0.78]; I^2^ = 94.30%), 0.90 (95% CI [0.83, 0.96]; I^2^ = 60.02%), and 0.80 (95% CI [0.71, 0.88]; I^2^ = 85.86%), respectively. The I^2^ showed substantial heterogeneity among adherence rates for the three targeted diseases. Meta-regression found no difference in the pooled adherence rate in the intervention group across studies stratified by disease type, intervention duration, ITT status, age, or intervention setting. The *p*-values for the Begg’s test and Egger’s test were 0.62 and 0.13, respectively, denoting a lack of publication bias.

## Discussion

Individuals living with chronic disease must cope with a plethora of unique barriers to exercise (e.g., disease-specific symptoms, comorbidities, fatigue) that are irrelevant for healthy populations. The purpose of this systematic review and meta-analysis was to examine the potential differences in adherence and dropout rates of aerobic physical activity interventions among people with chronic disease. We hypothesized that cancer would have the lowest adherence rates, based upon the low percentage of those with cancer who follow aerobic physical activity recommendations and given the known long-term effects of cancer treatment [21]. Contrary to our hypothesis, no statistically significant differences were found between the three chronic diseases. In addition, we did not see differences in adherence rates between strictly clinic-based (74% [95% CI, 0.65, 0.82]) and home-based (80% [95% CI, 0.65, 0.91]) aerobic exercise programs. These findings suggest that home-based programs may be just as feasible and perhaps, equally engaging, as programs designed with more professional supervision in rehabilitation clinics and research settings.

Overall, adherence to the exercise prescription (e.g. meeting aerobic physical activity guidelines) was 77%. It is important to note that nearly half (43.3%) of adherence data reported herein was not based on ITT (calculations only used data from those who completed studies). Failure to adopt ITT methods can inflate primary outcomes in RCTs [26], although ITT status did not appear to contribute to substantive differences in adherence across chronic conditions. The results suggest adherence to the physical activity guidelines is highly feasible among chronic disease populations, in both clinic and home-based physical activity interventions.

Given the substantial heterogeneity in adherence rates across studies within each targeted sub-population, it seems worthwhile to consider new approaches for increasing continued exercise engagement among individuals who may have particular difficulty (falling well short of the average). For example, a precision behavioral medicine approach could begin to identify “red flags,” (scores indicating below-average functioning) to facilitate decisions about the appropriate type or timing of interventions. Such an approach can be used to inform supplemental strategies focusing on the interaction among disease symptomology with physical functioning (e.g., mobility limitations) and psychological functioning (e.g., self-efficacy beliefs), all of which can change, and contributes to variability in adherence. In support of this, Pedersen and Saltin (2006) found that in general, adherence to physical activity prescriptions among patients with chronic disease are more likely to occur when they are individualized to the patient, initially supervised, and include both aerobic and strength components [27]. Interestingly, Wong, McAuley, and Trinh (2018) reviewed physical activity program preferences among cancer survivors and found a wide variation of preferences, suggesting that tailored programs may optimize program adherence [28].

Although the studies herein provide greater insight into aerobic physical activity adherence and dropout rates among chronic disease populations, insufficient information is available to explain our null findings. Many studies have targeted exercise determinants, yet evidence has not pointed to a consistent set of factors associated with adherence to physical activity guidelines or exercise programs. For example, using a broad framework incorporating many social and ecological factors, Kampshoff et al. [29] found that only one’s past exercise experience was associated with adherence among cancer survivors. Few systematic reviews exist, focused on determinants of exercise adherence for people with CVD and diabetes. Daly et al. [30] identified demographic factors as well as perceived benefits and leisure-time physical activity to be associated with non-adherence among cardiac rehabilitation patients. However, the authors pointed out that there were many methodological limitations of the studies in their sample (particularly a lack of randomized controlled designs), making it unclear as to which factors are associated with adherence levels among people with CVD. Allen [31] conducted a review examining exercise adherence in populations with diabetes and found self-efficacy measures to be predictive of exercise initiation and maintenance. However, with most studies included in the review, exercise was not the primary outcome. Rather the primary interventions were self-care regimens that included an exercise monitoring component. The limited research in this area and the variability among studies that do exist make it difficult to assess the most robust determinants of physical activity adherence among chronic disease populations.

Although it is unclear which factors reliably contribute to exercise adherence, specifically among people living with chronic disease, some factors seem to be unrelated (exercise modality, location). For example, Yang et al. [32] compared the effectiveness of aerobic and resistance exercise in populations with diabetes and found no evidence that either modality resulted in more favorable health outcomes. Yang et al. concluded that instead of focusing on the most preferred type of exercise, there should be a greater emphasis on getting chronic disease populations to remain physically active. In two other reviews of cardiac rehabilitation patients’ program adherence, Anderson et al. [33] and Dalal et al. [34] found that home- and center-based programs were equally effective in improving health-related quality of life. Anderson et al. also found programs that included self-regulatory factors (i.e. self-monitoring, action planning) resulted in the greatest levels of program adherence. Together, our findings coupled with prior research underscore the problem of exercise adherence. Perhaps a more patient-centered perspective and targeting self-regulatory deficiencies and strengths may benefit people with chronic conditions more so than generic exercise interventions designed to overcome common barriers.

There are several limitations that should be considered when interpreting the results of this study and should be addressed in future research. First, the exercise prescriptions varied in duration, intensity and complexity. In addition, this study only targeted trials lasting at least three months (again, for consistency purposes, to align with established data regarding exercise program dropout). This resulted in only 30 studies being used for the review and limited our power to test potential moderating characteristics of sample attributes and trial design.

## Conclusion

The findings of this review suggest that among people with different chronic conditions participating in aerobic physical activity interventions, there is consistency in the extent to which they are non-adherent. The overall rate of adherence was 77% to the physical activity programs, and the dropout rate was 7%, suggesting that people with chronic conditions are capable of sustaining physical activity for 3+ months, under different degrees of supervision, at levels sufficient for health benefits. Future researchers and healthcare providers should continue to develop adherence-promotion strategies that account for the shared barriers across chronic disease populations, as well as the known variability within these sub-populations.

## Abbreviations

CI: confidence interval
CINAHL: Cumulative Index to Nursing and Allied Health Literature
CVD: cardiovascular disease
ITT: intention-to-treat
MET: metabolic equivalent task
PRISMA: preferred reporting items for systematic review and meta-analysis
PRISMA-P: preferred reporting items for systematic review and meta-analysis protocols
RCT: randomized controlled trial
